# 

**Published:** 1889-05

**Authors:** 


### BOOK NOTICES.

VICKS' ILLUSTRATED MONTHLY.—Rochester. HORTICULTURAL ART JOURNAL.—Rochester. OUR YOUNG FOLKS and BABYLAND.—Boston, still smile upon us from our exchange table, in all the splendor of these bright May days.

CENTURY MAGAZINE.—The April number of this excellent periodical had several timely articles, exquisitely illustrated, devoted to the first President and the first Congress convened in New York, and Inauguration Day 100 years ago. The May number is no less interesting, none better. The Century Co., N. Y. City.

YOUTHS' COMPANION comes out in an exquisite Easter dress, with contents full of interest and instruction for both old and young. Handsomely illustrated. Weekly Perry Mason & Co., Boston.

A NEW magazine for teachers, outside of the usual line of school journals, made its appearance May 1, 1889, with the endeavor to interest teachers and older pupils in the best attainments of the scientific, literary, and civil world, and present an "outlook" upon current events, etc. It is called the TEACHER'S OUTLOOK, and published by the Teachers' Publishing Co., Des Moines, Iowa.

AMERICAN RESORTS, with notes upon their climate, is a new volume by Bushrod W. James, A. M., M. D., intended for invalids and health seekers. The work has a wide reach, embracing information of value concerning the prominent sea shore, lake, springs and mountain resorts of North and South America, with a treatise translated from the German, relating to their varied climates. The book is of great interest to those in search of diversion and health. F. A. Davis, publisher, New York, Philadelphia, San Francisco and London.

DAHLONEGA (Ga.) *Signal* says : "A good many people are accustomed to use Peach-tree Bark Tea when sick. So we will give them something new—at least to us—which several good citizens have vouched for as the truth : When tea is made from bark that is skinned off upward, the tea acts as an EMETIC ; when skinned downward, it acts as a CATHARTIC."

